# Data on B cell phenotypes in baboons with pig artery patch grafts receiving conventional immunosuppressive therapy

**DOI:** 10.1016/j.dib.2018.08.213

**Published:** 2018-09-13

**Authors:** Takayuki Yamamoto, Qi Li, Hidetaka Hara, Liaoran Wang, Hongmin Zhou, Juan Li, Devin E. Eckhoff, A. Joseph Tector, Edwin C. Klein, Ray Lovingood, Mohamed Ezzelarab, David Ayares, Yi Wang, David K.C. Cooper, Hayato Iwase

**Affiliations:** a Xenotransplantation Program, Department of Surgery, University of Alabama at Birmingham, Birmingham, AL, USA; b Second Affiliated Hospital, University of South China, Hengyang City, Hunan, China; c Department of Cardiothoracic Surgery, Tongji Medical College, Huazhong University of Science and Technology, Wuhan, China; d Thomas E. Starzl Transplantation Institute, University of Pittsburgh, Pittsburgh, PA, USA; e Kirklin Clinic Pharmacy, University of Alabama at Birmingham, Birmingham, AL, USA; f Revivicor, Blacksburg, VA, USA

## Abstract

This report is related to the research article entitled “B cell phenotypes in baboons with pig artery patch grafts receiving conventional immunosuppressive therapy” (Yamamoto et al., in press). Herein we provide the data regarding pig artery patch xenotransplantation into the baboon׳s aorta, trough levels of tacrolimus and rapamycin in the blood after transplantation, analysis of B cell phenotype on the basis of IgD and CD27 expression in the blood, and analysis of T cell phenotype on the basis of CD28 and CD95 expression in the blood.

**Specifications table**

**Table:** 

Subject area	*Medicine, Immunology*
More specific subject area	*Transplantation*
Type of data	*Table, image, text file, figure*
How data was acquired	*Immunological assays, animal experiments*
Data format	*Analyzed data*
Experimental factors	*Pig artery patch graft in immunosuppressed baboons*
Experimental features	*Pig artery patch transplant in baboons, immunomonitoring*
Data source location	*University of Alabama at Birmingham, Birmingham, AL, USA*
Data accessibility	*Data are included in this article*

**Value of the data**

● These data provide methods and analysis of investigating B cells and T cells in xenotransplantation.
● These data describe B cell and T cell monitoring in the pig-to-baboon artery patch model.
● These data provide information on the efficacy of FDA-approved immunosuppressive agents in xenotransplantation.

## 1 Data

### 1.1 Pig artery patch graft in baboon aorta

**Fig. 1:**
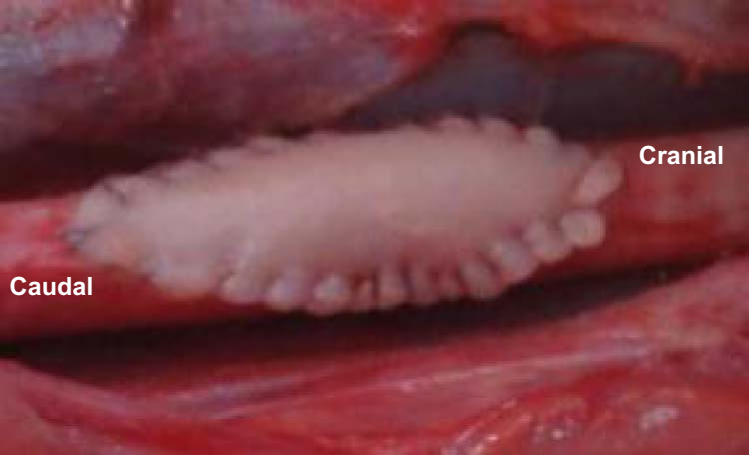
Pig artery patch graft in baboon׳s aorta.

Fig. 1 illustrates the surgical technique in this model.

### 1.2 Trough levels of tacrolimus and rapamycin in the blood (Fig. 2)

**Fig. 2:**
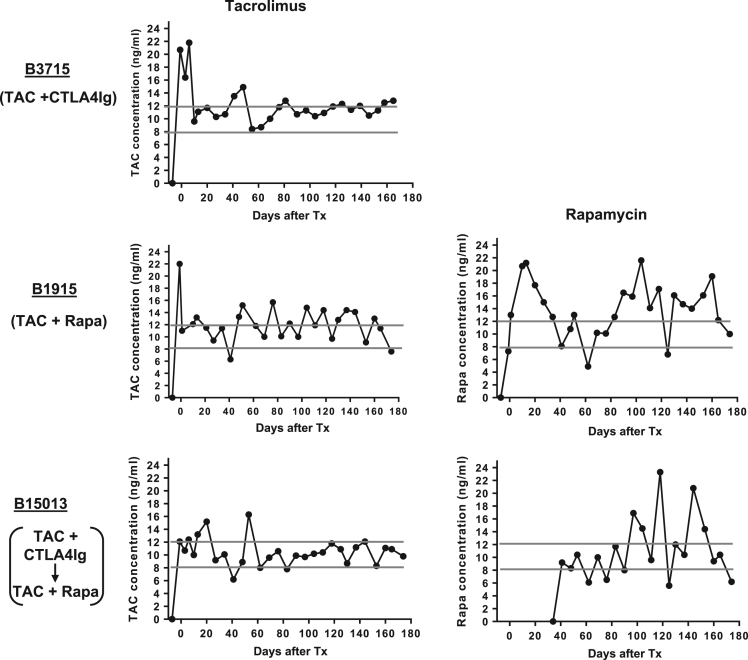
Trough levels of tacrolimus and rapamycin in the blood. The target trough level of both drugs was 8–12 ng/ml. Gray bars represents the target range. (Abbreviations: TAC = tacrolimus; Rapa = rapamycin).

The target trough levels of tacrolimus (TAC) and rapamycin (Rapa) were both 8–12 ng/ml. Mean (±SD) tacrolimus trough levels were 12.24 ± 0.60 ng/ml (B3715), 12.16 ± 0.58 ng/ml (B1915), and 10.55 ± 0.40 ng/ml (B15013). Mean (±SD) rapamycin trough levels were 13.76 ± 0.85 ng/ml (B1915) and 11.19 ± 1.06 ng/ml (B15013) (Fig. 2).

### 1.3 Lymphocyte, T and B cell counts after transplantation (Table 1)

Lymphocyte counts in B3715 gradually increased from 1 m after transplantation, and those in B1915 increased after 2 m. However, those of B15013 remained low throughout the 6 m period of follow-up. CD3^+^T and CD22^+^B cell numbers followed similar trends (Table 1).

**Table 1:** Lymphocyte, T cell, and B cell counts after transplantation.

**Lymphocytes (/mm**^**3**^**)**							
	**pre Tx**	**1M**	**2M**	**3M**	**4M**	**5M**	**6M**
**B3715**	1254	187	336	522	700	1064	1176
**B1915**	1680	96	155	361	456	660	598
**B15013**	1189	140	216	230	239	240	240

**CD3**^**+**^**T cells (/mm**^**3**^**)**							
**B3715**	993	108	248	340	487	644	680
**B1915**	1278	72	109	261	245	346	255
**B15013**	1086	114	173	185	229	193	176

**CD22**^**+**^**B cells (/mm**^**3**^**)**							
**B3715**	182	2	15	99	125	164	181
**B1915**	272	1	1	11	88	162	183
**B15013**	151	2	5	12	22	12	14

### 1.4 Analysis of B cell phenotype on the basis of IgD and CD27 expression in the blood of an immunologically-naïve baboon (Fig. 3)

**Fig. 3:**
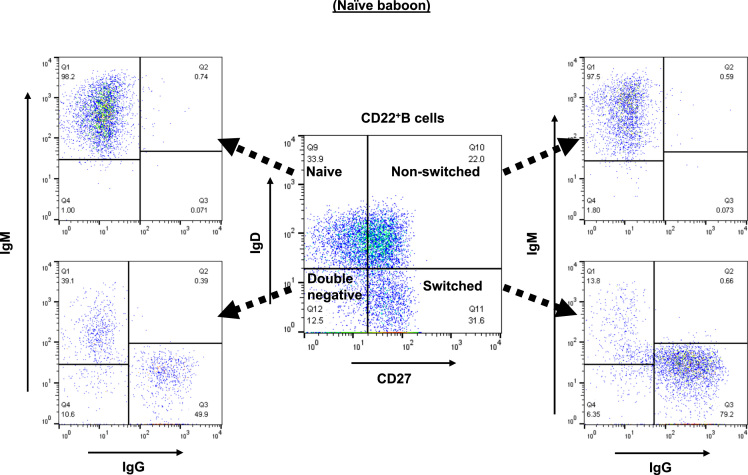
Analysis of B cell phenotype on the basis of IgD and CD27 expression in the blood of an immunologically naïve baboon.

CD22^+^B cell memory phenotypes in the blood were determined on the basis of IgD and CD27 expression by flow cytometry. CD3^−^CD22^+^B cells were classified as IgD^+^CD27^-^ naïve (which express predominantly IgM), IgD^+^CD27^+^ as non-switched memory (which express predominantly IgM), IgD^-^CD27^+^ as switched memory (which express predominantly IgG), and IgD^-^CD27^-^ as double-negative (which express both IgM and IgG) (Fig. 3).

### 1.5 Dynamics of repopulating B cell phenotypes after transplantation (Fig. 4)

**Fig. 4:**
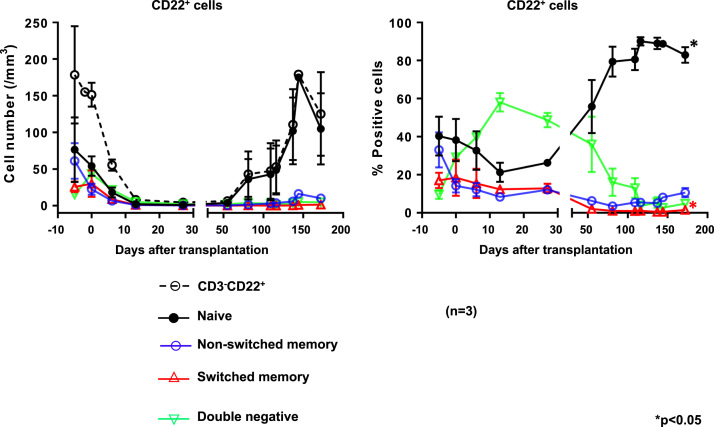
Dynamics of repopulating B cell phenotypes after transplantation. (Left) The Y axis represents cell numbers. (Right) The Y axis represents percentage of each cell. *p < 0.05.

The percentage of naïve memory B cells increased significantly in all baboons (pre-transplant [day -5] = 33.60 ± 6.48%; post-transplant [at 6 m] = 88.97 ± 2.99%, *p* = 0.0015). In contrast, there was a significant decrease in switched memory B cells (pre-transplant [day -5] = 17.07 ± 4.03%; post-transplant [at 6 m] = 0.53 ± 0.28%, *p* = 0.015) (Fig. 4).

### 1.6 T cell responses in pig artery patch recipients

**Fig. 5:**
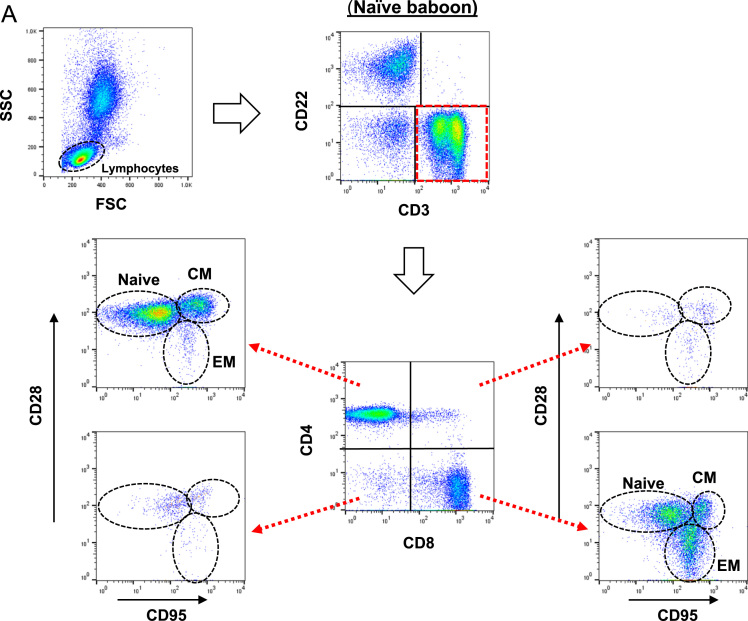

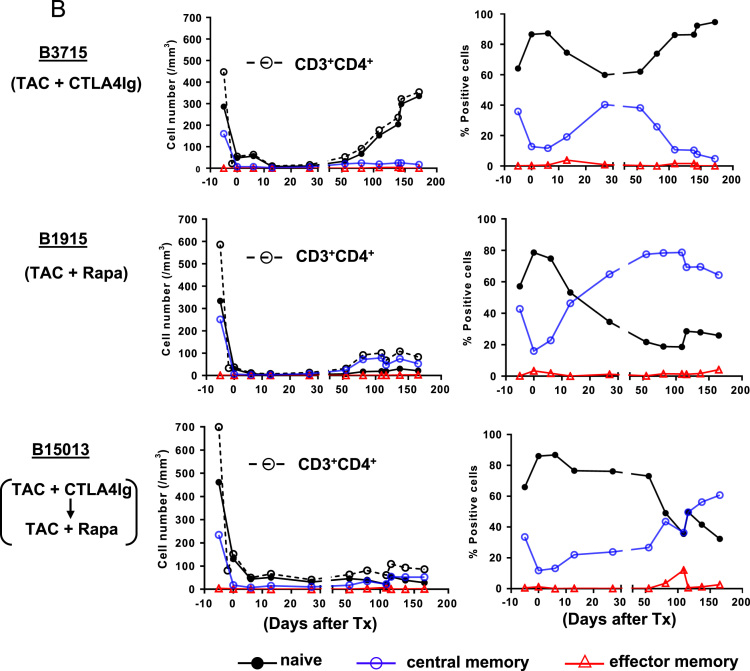

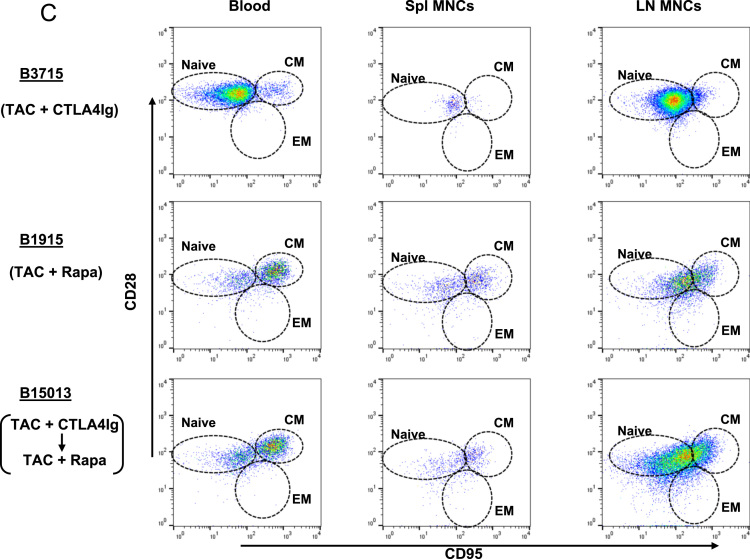

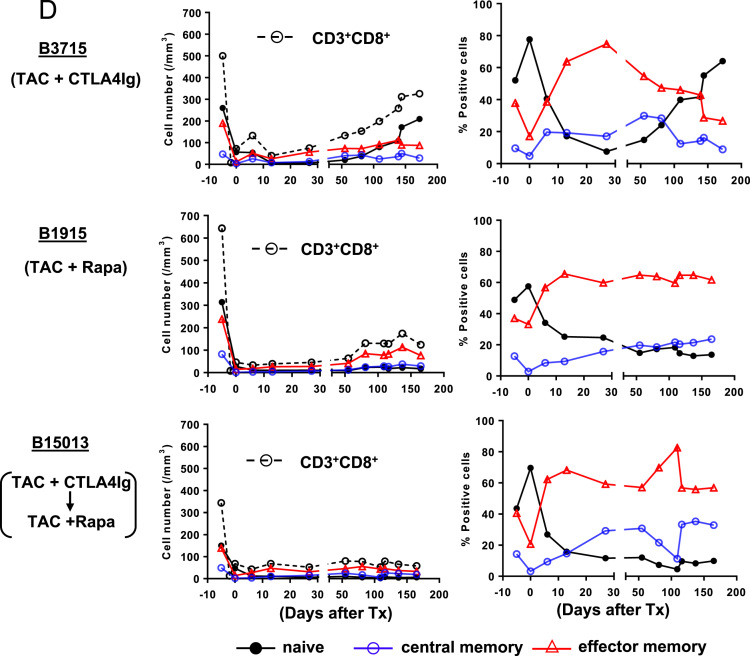

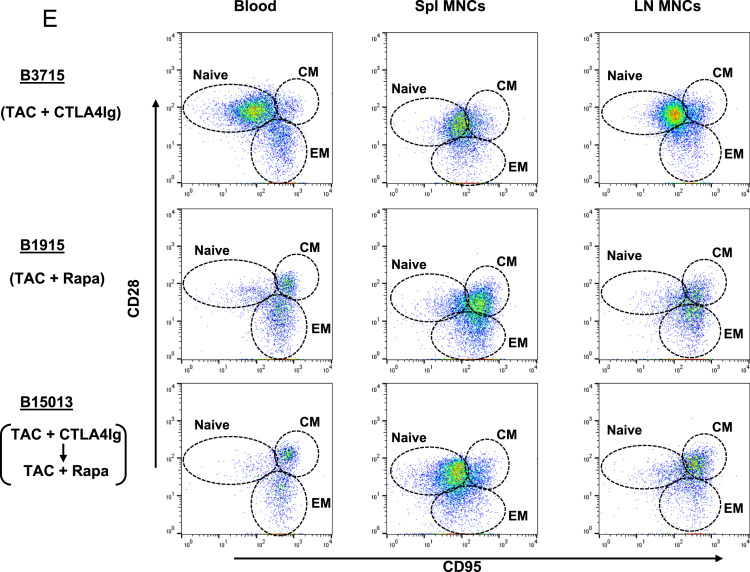
T cell responses in pig artery patch recipients. (A) Analysis of T cell phenotype on the basis of CD28 and CD95 expression in the blood of a naïve baboon. (Abbreviations: CM = central memory; EM = effector memory). (B) Dynamics of repopulating CD4+T cell phenotypes after transplantation. (Left) Y axis represents cell numbers. (Right) Y axis represents percentage of each cell. (Abbreviations: TAC = tacrolimus; Rapa = rapamycin). (C) CD4+T cell phenotype in blood and secondary lymphoid tissues (at euthanasia 6 m after transplantation). (Abbreviations: TAC = tacrolimus; Rapa = rapamycin; CM = central memory; EM = effector memory; LNMNCs = lymph node mononuclear cells; SplMNCs = spleen mononuclear cells.). (D) Dynamics of repopulating CD8+T cell phenotypes after transplantation. (Left) Y axis represents cell numbers. (Right) Y axis represents percentage of each cell. (Abbreviations: TAC = tacrolimus; Rapa = rapamycin). (E) CD8+T cell phenotype in blood and secondary lymphoid tissues (at euthanasia 6 m after transplantation). (Abbreviations: TAC = tacrolimus; Rapa = rapamycin; CM = central memory; EM = effector memory; LNMNCs = lymph node mononuclear cells; SplMNCs = spleen mononuclear cells).
  Using multicolor flow cytometry, we distinguished CD3^+^CD4^+^ or CD3^+^CD8^+^T cells into different subsets on the basis of CD28 and CD95 expression. CD28^+^CD95^-^ were classified as naïve cells, CD28^+^CD95^+^ as central memory cells, and CD28^-^CD95^+^ as effector memory cells.
(B) *Dynamics of repopulating CD4*^*+*^*T cell phenotypes after transplantation* (Fig. 5B)
  These data include CD4^+^T cell numbers before immunosuppressive therapy was initiated (control). In B3715, a naïve phenotype persisted. In contrast, B1915 and B15013 showed gradually increasing central memory phenotypes, especially with regard to the percentage of positive cells. In B15013, the increase did not begin until 50 days after transplantation.
(C) *CD4*^*+*^*T cell phenotype in blood and secondary lymphoid tissues (at euthanasia 6 m after transplantation)* (Fig. 5C)
  (LNMNCs = lymph node mononuclear cells; SplMNCs = spleen mononuclear cells.) CD4^+^T cells in B3715 in the blood and secondary lymphoid tissue (SplMNCs and LNMNCs) 6 m after transplantation were mostly of the naïve T cell phenotype. In the other 2 baboons (B1915 and B15013), the CD4^+^T cells in the LNMNCs 6 m after transplantation were mostly of the naïve T cell phenotype; however, in the blood, the CD4^+^T cells were mostly of the central memory T cell phenotype.
(D) *Dynamics of repopulating CD8*^*+*^*T cell phenotypes after transplantation* (Fig. 5D)
  These data include CD8^+^T cell numbers before immunosuppressive therapy was initiated (control). B3715 showed an increasing effector memory phenotype 1 m after transplantation, followed by a gradually recovering naïve phenotype. In contrast, B1915 and B15013 showed immediately increasing effector memory phenotypes, especially with regard to the percentage of positive cells.
(E) *CD8*^*+*^*T cell phenotype in blood and secondary lymphoid tissues (at euthanasia 6 m after transplantation)* (Fig. 5E)
  CD8^+^T cells in B3715 in the blood and secondary lymphoid tissue (SplMNCs and LNMNCs) 6 m after transplantation were mostly of the naïve T cell phenotype. The CD8^+^T cells in B15013 in SplMNCs 6 m after transplantation were mostly of the naïve T cell phenotype. However, the CD8^+^T cells in B1915 and B15013 in the blood and LNMSCs 6 m after transplantation were mostly of the central and effector memory T cell phenotypes.

(A) *Analysis of T cell phenotype on the basis of CD28 and CD95 expression in the blood of a naïve baboon* (Fig. 5A)

## 2 Experimental design, materials and methods

### 2.1 Pig-to-baboon artery patch xenotransplantation

Details are provided in our research paper [1] and in a previous paper [2].

### 2.2 Immunosuppressive, anti-inflammatory, and supportive therapy

Details are provided in our research paper [1].

### 2.3 Monitoring of recipient baboons

Details are provided in our research paper [1] and in previous papers [3], [4], [5], [6].

#### 2.3.1 Flow cytometry

Lymphocyte subsets were distinguished by mAbs to surface antigens. Baboon blood PBMCs, SplMNCs and LNMNCs (100 µl) were incubated with Alexa Fluor 700-conjugated anti-human CD3 (clone SP34-2), fluorescein isothiocyanate (FITC)-conjugated anti-human CD4 (clone L200), phycoerythrin (PE)-Cy7-conjugated anti-human CD8 (clone RPA-T8), allophycocyanin (APC)-H7-conjugated anti-human CD20 (clone 2H7), peridinin-chlorophyll proteins (PerCP)-Cy5.5-conjugated anti-human CD28 (clone CD28.2) and APC-conjugated anti-human CD95 (clone DX2) antibodies (all from BD Pharmingen, San Diego, CA), PE-conjugated (clone RFB-4) anti-human CD22 antibody (Invitrogen, Carlsbad, CA), PerCP-Cy5.5-conjugated (clone O323) anti-human CD27 antibody (iCyt, Champaign, IL) and FITC-conjugated goat anti-human IgD antibody (SouthernBiotech, Birmingham, AL). Incubation was for 30 min in the dark at 4 °C. After setting compensation and gating correctly, at least 50,000 events were acquired. Specimen acquisition was performed using LSR II flow cytometer (Beckton Dickinson, Franklin Lakes, NJ) and the obtained data were analyzed with Flowjo V10 (Tree Star, Ashland, OR).

The percentages of lymphocytes, B cells, T cells, and other cells were measured by flow cytometry, and the absolute counts of WBCs, lymphocytes, monocytes, and granulocytes were measured by standard methods (ANTEC, Birmingham, AL).
