# Supplementary material for: Data on B cell phenotypes in baboons with pig artery patch grafts receiving conventional immunosuppressive therapy
Source: Data Brief. 2018 Sep 13;20:1965–74. doi: 10.1016/j.dib.2018.08.213 (PMC6171326; doi:10.1016/j.dib.2018.08.213)
Supplement: Supplementary file 1 — Supplementary material. [file mmc1.docx]

**DISCLOSURE OF CONFLICT OF INTEREST**

David Ayares is an employee of Revivicor, Inc. No other author has a conflict of interest.
